# Strategic emergency department design: An approach to capacity planning in healthcare provision in overcrowded emergency rooms

**DOI:** 10.1186/1752-2897-2-11

**Published:** 2008-11-17

**Authors:** Aristomenis K Exadaktylos, Dimitrios S Evangelopoulos, Marcel Wullschleger, Leo Bürki, Heinz Zimmermann

**Affiliations:** 1 Department of Emergency Medicine, Inselspital-University Hospital, Bern, 3010 Bern, Switzerland; 2 Department of Orthopaedic Surgery, Inselspital-University Hospital, Bern, 3010 Bern, Switzerland; 3 Department of teaching and research, Inselspital-University Hospital Bern, 3010 Bern, Switzerland; 4 SYSTEMIK & Sustainability Managementzentrum Bern, Fachhochschule Bern HTI, Switzerland; 5 Department of Emergency Medicine, Inselspital-University Hospital Bern, 3010 Bern, Switzerland

## Abstract

Healthcare professionals and the public have increasing concerns about the ability of emergency departments to meet current demands. Increased demand for emergency services, mainly caused by a growing number of minor and moderate injuries has reached crisis proportions, especially in the United Kingdom. Numerous efforts have been made to explore the complex causes because it is becoming more and more important to provide adequate healthcare within tight budgets. Optimisation of patient pathways in the emergency department is therefore an important factor.

This paper explores the possibilities offered by dynamic simulation tools to improve patient pathways using the emergency department of a busy university teaching hospital in Switzerland as an example.

## Background

In the USA and Europe, emergency departments (EDs) are confronted with overcrowding and budget restrictions. The discipline of emergency medicine (EM) has faced significant challenges from its inception to its successful establishment in many countries. Inherent to EM is the need to interact with many other specialties, and the results have been rewarding, disappointing, or questionable to almost equal extents. One of the most consistent frustrations for EM physicians are constantly overcrowded emergency rooms. The profile of the patient population is also changing and is highly dependent on cultural context and developments. Furthermore, there is an increasing need for more information on financial planning and health policy-making [1]. These factors provide a stimulus to analyze and improve internal processes in EDs. Traditionally, such design-relevant problems were solved by means of static (quantitative) estimates, but the use of a qualitative dynamic systems (DS) approach seems to be more appropriate [2].

The ED at the Inselspital in Berne, Switzerland, provides round-the-clock medical and surgical care. An increasing patient volume has resulted in difficulty in reaching elective admission and bed occupancy targets. We used data from our department to map the system conceptually based on patient pathways from admission to discharge. Patterns of activity, demand, and system bottlenecks were simulated with this map and used to construct a quantitative DS model. Our goal was to examine whether a DS model approach can help to solve strategic design challenges in emergency department capacity planning, and demonstrate the significance of such a design feature in achieving strategic and political success.

## Methods

Our goal was to solve complex problems within the framework of a process owner (the ED), a strategy owner (hospital management) and a model owner (systemic consultant) by implementing new ways of working together for all concerned in the hospital environment. It was also important to demonstrate the usefulness of the DS approach by collecting key data and transforming it from a static view into a dynamic understanding of a situation.

The collection of specific data and key information is strongly recommended in the context of ED design [1]. Although indispensable, data such as annual patient volumes, patient acuities, and patient age distribution give only a static system view.

Contrary to the USA, European EDs are split into surgical and medical divisions or tracks, depending on the hospital. An incoming patient is initially treated in a treatment berth and then leaves the ED as an outpatient, or is transferred to a ward. Patients with non-urgent conditions or attending for follow-up examinations are treated in the 'fast track' or ambulatory clinic. The different units can be considered as subsystems within the ED.

**Figure 1:**
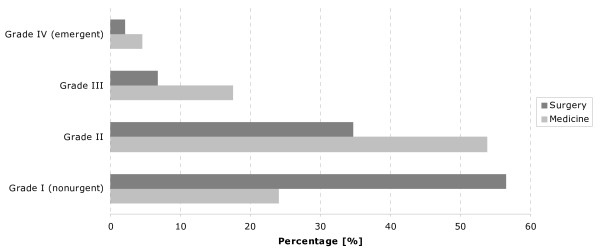
Distribution of acuity from grade I (non-urgent) to grade IV (emergent) in the surgical and medical tracks.

Depending on the particular subsystem (ambulatory, treatment berth, bed), outpatient and inpatient distribution differs between the medical (14.3% outpatients versus 19.3% inpatients) and surgical (36.7% outpatients versus 6.5% inpatients) track or unit. Figure 1 depicts the acuity distribution for a certain period of time: there are more medical patients with higher acuity than surgical patients. We therefore assumed that more inpatients than outpatients have a higher average acuity in the medical unit than in the surgical unit.

Acuity has 4 grades or levels of severity. Acuity is defined on admission and not after full medical evaluation. Discrepancies between the assessment of acuity by the patient and physician are especially common in the non-urgent category, particularly when variables such as environmental factors (i.e. waiting time) are taken into account.

### Static system view

Precise definition of 'simple' input parameters is the best means of obtaining comparable information in the long term. It is important that all stakeholders agree on these definitions. We used the following:

• The *ED patient *was defined (in the static sense where data is concerned) as a monthly 'identity' (i.e. one identity is at least one patient per month).

• The *ED case *was defined by its unique number.

• An *ED visit *was defined as any admission to the ED, regardless of patient identity or case number (i.e. a patient can have one or more visits corresponding to the same or to a different case).

**Figure 2:**
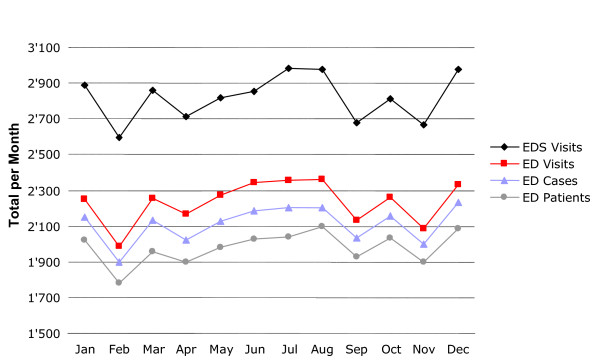
The *ED visits *is the number of patient visits to the emergency department in a certain period. The *EDS visits *are the sum of the visits to the subsystems (ambulatory system, treatment berth system, bed system) within the ED in a certain period. The EDS visits figure is higher than the ED visits figure due to the cumulative effect (the same patient may pass through different subsystems within one patient process). The difference between the two figures may provide additional information on workload. Similar to ED visits, we distinguish between ED cases (number of cases of illness) and ED patients (personal identity).

Similarly we have the ED subsystems (*EDS) visits *(including the ambulatory system [AS], the treatment berth system [TBS], and the bed system [BS]). Because incoming patients are initially difficult to categorize, an outpatient can shift to inpatient status, or vice versa. The statistic *EDS visits *provides an internal static view. Static data such as totals and averages may be required for statistical analyses but they *do not provide sufficient information for capacity planning*. Static numbers cannot reflect the distribution of patient admissions over a certain period. Numbers are only comparable if the situations from which they are derived are also comparable (Figure 2).

### Dynamic system view

Unlike static information, a dynamic view allows peak volumes to be highlighted and can simulate capacity shortages as perceived by staff. Capacity planning is normally based on averages, but peak numbers of patients in the ED (or EDS) at key times per day and month [2] are also of great relevance.

Static information may be linked to qualitative parameters [2]. Simulation 'activates' the simple static ED statistics. In other words, in the following, the term 'patient' (i.e. number of patients in the ED) means the dynamic view with patients who actually appear (i.e. at key times), and is therefore based on the 'activated' ED visit figure. The dynamic view gives a picture of increased complexity (individual length of stay, non-linear distribution of entry and exit rates), but which is much closer to reality and provides relevant information for capacity planning.

### Dynamic system simulation

DS simulation offers decision-makers an accessible, cheaper, and timelier means of evaluation [3], improvement and optimisation of several processes. It imitates an actual process over time [4]. Simulation models imitate a system's behaviour, referred to as 'baselining', and are then used to evaluate possible changes in structure and environment by incorporating underlying assumptions in the form of a 'what-if' analysis [4].

We used *Ithink *software by *isee systems *(Lebanon, NH 03766, USA; formerly High Performance Systems, HPS) [5]. *isee *systems has become the world leader in Systems Thinking software based on the DS approach [6]. It therefore seemed an appropriate approach to assist us in answering our questions, because it offers state-of-the-art simulation of complex feedback systems such as hospital environments. Real-time simulations are performed on this defined methodical base, and users have only to deal with modelling reality into processes. Once a model has been built, it is easy to create a high-quality user interface, simulate different decision scenarios, and view the consequent behaviours.

The system's inbuilt RND function returns a single number, 'randomly' chosen between 0 and 1 (in the language of probability, the number is uniformly distributed between the range of 0–1). A linear congruential generator (LCG) is based on the formulae developed by Lehmer (1948): {rnd(i+1) = (rnd(i)*b+a) *mod *max}. *Ithink *is based on LCGs and these are currently used in almost all random number generators.

To simulate the different events, discrete simulations of patients use the following statistical methods: ED entrances are randomised by Poisson distribution, varying along the day line, as described in this article. For triage to different parts of the ED, normal distribution was extrapolated from statistics. Duration of stays in berths or fast-track beds were simulated by exponential distribution to simulate realistic behaviour, as recommended in standard literature and DS standards.

When developing a DS model with *Ithink *software the following five steps are important:

#### 1. Goals and objectives of the simulation

The simulation should generate information that can be used by management to make appropriate decisions, solve capacity problems of the ED(S), install a planning process, and obtain a convincing and trusted 'dynamic decision-making tool' for best practice.

It is also important to exclude goals that cannot be reached by simulation and to be aware that simulation also cannot advise on the kind of scenarios to test. This is the responsibility of the team and decision-makers, and depends on their understanding of the whole complex process. What DS does is to supply answers or indicate trends, by testing ideas with different scenarios [7].

#### 2. Describe the current system and development of the model

The framework for the ED(S) model was developed to map patient flow. The process owners of the ED confirmed the patient flow diagram. This model was developed to replicate current daily business and to communicate current bottlenecks in the ED(S). The level of detail chosen for a model is extremely important in achieving useful results. As the model becomes more complex, it requires additional data and continuous testing.

#### 3. Collecting data

**Figure 3:**
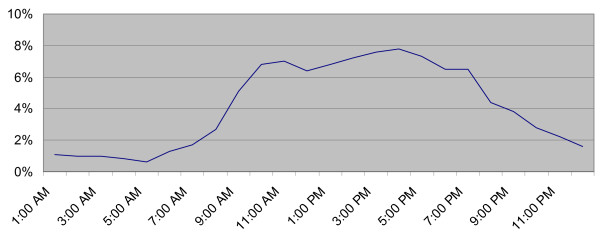
Data required for the simulation: proportional distribution of patient admissions over 24 hours. Data was collected for three months: number of surgical, medical and fast track patients.

Simulation needs well-defined core data collected from the ED. We differentiated between the number of ED visits in the simulation using the time of patient arrival. The 24-h patient admission distribution (Figure 3) in combination with an average daily amount of patient arrivals is important to simulate realistic daily fluctuation. Figure 3 assists in obtaining a realistic simulation of the bottlenecks, because the *patient flow *strongly fluctuates and defines the capacity needed, which cannot be achieved with statistical analysis of average estimates. The patient process analysis has to incorporate a description of key subprocesses during normal patient flow and in extreme daily behaviour (capacity and mean time). To arrive at solutions for problems with patient processes, e.g. decreasing patient waiting times and optimum resource utilization, we no longer describe capacity, organization and quality management by using only statistics, and these aspects have to be reviewed by simulating different scenarios.

**Figure 4:**
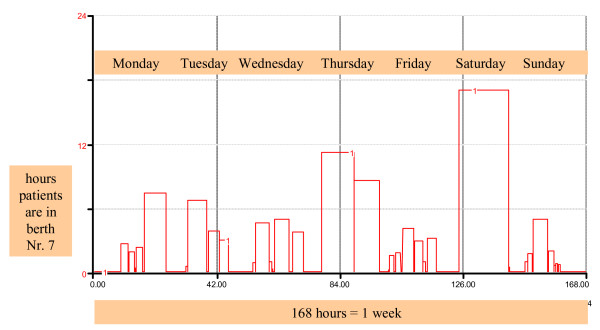
Example of the occupation pattern of ED treatment in a berth No. 7 over one week: the x-axis shows one week (hours) and the y-axis indicates the hours of occupation. Each berth is simulated separately in real-time. The pattern of occupation of all berths must be as realistic as possible to gain maximum benefit in the scenarios developed by the process owners.

The dynamic effects of all subprocesses have to be reviewed, such as the sub-process 'simulated pattern of use for ED treatment berth No. 7' hourly throughout one week, as shown in Figure 4.

#### 4. Testing the model

The key data are visualized in a 'flight simulator' interface, and graphic displays show the pattern of a week in the ED(S). The process owners are able to see at a glance whether the model will bring any advantages by comparison of the number of patients waiting in the ED(S) area or the fluctuations in the numbers of incoming patients.

#### 5. Systems thinking-scenarios, analyses and solutions

Ideas and solutions for alternative approaches can be evaluated after testing the simulation model. Since simulation models evaluate outcomes without making changes to the real system, simulation modelling permits the evaluation of different alternatives before any resources are expended. The accountability for the variation in patient arrival times, utilization of infrastructure, queuing, and treatment times are vital for results in a process that is dominated by interaction between human beings [8].

## Results

### Scenario 'Business as usual' – by annual growth + 5% and shift to more medical patients

**Figure 5:**
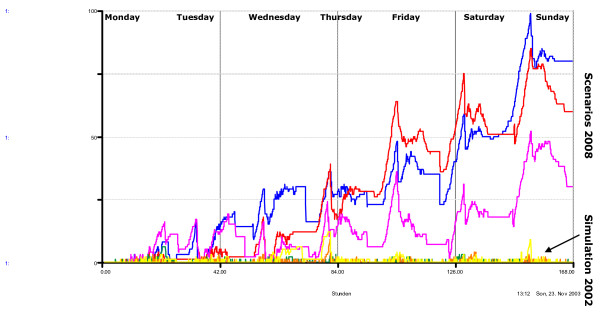
The graph represents the number of *beds lacking *at a given capacity (BS) during one week for different scenarios; it shows a 2002 simulation (yellow, orange, green) and 3 comparative scenarios in 2008 (blue, red, pink). The scenarios were varied according to adjustable variables such as patient volume, patient mix (i.e. acuity, surgery vs. medicine), arrival time distribution, and average length of stay.

The simulation depicted in Figure 5 shows impressively how continuous growth of patient flow results in overproportional growth in patient waiting times. The management of the TBS and BS loses all its flexibility: a simulation for 2002 (30,000 patients; 56% surgery/44% medicine) showed that there is sometimes a lack of available beds, but only for a short time. In contrast, the scenario for year 2008 (35,000 patients; 53% surgery/47% medicine) showed an increasing lack of beds in three comparative simulations. As mentioned above, the growing number of patients in our ED is mostly due to the more sensitive non-urgent patient. This may damage the image of an ED by causing long waiting times, which are well-recognized as detrimental: "Like it or not – it's the key to your hospital's reputation. Three times as many patients create their impressions of your hospital through the ED than by admissions and 30% to 40% of admissions come through the ED" [1].

The scenarios described above force management to act: if there are no strategy-based changes (i.e. in triage or collaboration with other providers), structural and spatial (operative) planning of the ED(S) will face serious problems. Trends, which are regarded as neither good nor bad, should be strategically evaluated, communicated, and suitable corrective action taken, if necessary.

It is estimated that more than 75% of reengineering efforts do not produce target performance improvements; and hospitals supply vivid testimony to the fact that growth strategies often fail to yield real growth. Stories abound of costly organizational change efforts that either changed nothing, or worse still, exacerbated the situations they aimed to improve. The ED(S) simulation model represents the actual situation. Thus it is important consider how our performance initiatives come into being. The simple answer is that very often they are just 'thought up'. We work with our mental model of reality, which is actually a subjective abstraction of reality. If dynamic methods are used, even process owners are often surprised about the non-linear behaviour of complex systems where a small impact can sometimes have very large consequences.

### Scenario ED Treatment Berth System vs ED Bed System

ED(S) distribution of patients in the TBS and the BS is a result of daily business development and adapts to available facilities. The ED(S) representatives were convinced that more bed capacity in the BS would be the best strategy. This idea was also fed through the newly discussed strategy to stop transferring patients to the ward between 17:00 and 08:00 (i.e. keeping patients in the BS over night). Testing of different scenarios convinced the process owners that they would gain more flexibility and incur lower costs by increasing treatment berths. Analysis of the pattern of patient arrivals showed that bed capacity was not crucial during the night, as they had thought. They therefore requested more treatment berth capacity and not an increase in the number of beds.

To enable new ideas of this sort, the variables must be able to be adjusted in any constellation. One of the major strengths of DS is that different scenarios and results are analysed from different points of view by all stakeholders.

### Reaction to the trend of increasing numbers of 'self-declared non-urgent EM patients'

Waiting times in EDs are a significant problem and are made more complex by 'self-declared non-urgent patients'. The patient's length of stay in an ED varies considerably, and this makes effective management of an ED difficult. The authorities' response to growing patient waiting times was to implement a fast track, where a specifically trained nurse performed the triage, and the non-urgent patients were sent directly to the fast track. The new situation was easy to map in the model, but the results did not correspond to reality. Simulation showed that with the available infrastructure, it would not be possible to maintain medium waiting time below one hour. The process owners, however, had the impression that the waiting time was much less in reality. The simulation model allowed only one item to be adjusted, which explained this contradiction, but the results were nevertheless paradoxical: after the triage of non-urgent emergency patients, the more severely injured patients stayed in berths and beds for shorter periods. New data from the IT system were evaluated and confirmed this: the length of stay was reduced by 30 minutes to 60 minutes.

The process owners commented that they have better IT solutions, better management and more employees. Previously, patients passed thorough a sequence of independent steps, such as signing in, filling out a history, waiting for a triage nurse, noting vital signs. Establishing the fast track brought about a general shift in organization. The triage process was simplified by starting assessment at the time of patient arrival. Now, at entry, the triage nurse made the first contact with the patient and immediately contacted the physicians. This was a first step from sequential to parallel processing in ED(S), and higher patient satisfaction and shorter stays in ED(S) will be the result of the complete shift to parallel processing. This example shows that retrospective analysis of simulations can also lead to improvement. The next step might be scenarios of different parallel processing models to obtain even better results.

Better management ultimately led to lower infrastructural demands, as had been requested at the beginning of the planning process.

### Lessons learned for the planning process

Forward-looking decision-making processes have far-reaching consequences for the healthcare sector. Decision-makers must know what they are doing, why they are taking certain measures, and what action has to be taken to achieve a satisfactory outcome. Overall, there is a growing awareness of the need to ensure viability and effectiveness of healthcare services, particularly emergency services. To achieve this, it is necessary to create three complementary processes [10]:

▪ System knowledge: the process owners define the problems

▪ Transformation knowledge: the modellers bring in synergies for simulation scenarios

▪ Objective knowledge: the strategic planners define roles and new fields

Different models and techniques, such as the Markov Model and Neuronal Network Models are used to assist with redesigning hospital management. Neuronal networks have been extensively studied as computational systems, but they also serve as communications networks in transferring large amounts of information [11,12]. Their structure and function are governed by basic principles of resource allocation and constraint minimization [13]. Some of these principles could be incorporated into simulations like ours.

Our simulation model was useful for investigating specific emergency room scenarios in terms of patient flow and bottlenecks, and, perhaps more importantly, as a device for provoking and facilitating discussion and comment amongst all those concerned in the care of acutely ill patients. It was not intended to be an exclusive solution for dynamic simulations in medicine. The team involved readily accepted that the model gave only an indication of the relative effects of different interventions, rather than mathematically precise forecasts or point predictions, and were enthusiastic to suggest alternative scenarios for testing based on earlier simulations.

## Competing interests

The authors declare that they have no competing interests.

## Authors' contributions

AE wrote and revised the manuscript, DSE wrote and revised the manuscript, MW and LB provided technical information, essential in the planning of the manuscript, HZ planed, wrote and supervised the manuscript.
